# Theoretical Study of the Magnetic and Optical Properties of Ion-Doped Li*M*PO_4_ (*M* = Fe, Ni, Co, Mn)

**DOI:** 10.3390/ma17091945

**Published:** 2024-04-23

**Authors:** Iliana N. Apostolova, Angel T. Apostolov, Julia Mihailowa Wesselinowa

**Affiliations:** 1 University of Forestry, Kl. Ohridsky Blvd. 10, 1756 Sofia, Bulgaria; inaapos@abv.bg; 2 University of Architecture, Civil Engineering and Geodesy, Hr. Smirnenski Blvd. 1, 1046 Sofia, Bulgaria; angelapos@abv.bg; 3 Faculty of Physics, Sofia University “St. Kliment Ohridski”, J. Bouchier Blvd. 5, 1164 Sofia, Bulgaria

**Keywords:** Li*M*PO_4_, ion doping, magnetization, band gap, microscopic model

## Abstract

Using a microscopic model and Green’s function theory, we calculated the magnetization and band-gap energy in ion-doped Li*M*PO_4_ (L*M*PO), where *M* = Fe, Ni, Co, Mn. Ion doping, such as with Nb, Ti, or Al ions at the Li site, induces weak ferromagnetism in LiFePO_4_. Substituting Li with ions of a smaller radius, such as Nb, Ti, or Al, creates compressive strain, resulting in increased exchange interaction constants and a decreased band-gap energy, $E_{g}$, in the doped material. Notably, Nb ion doping at the Fe site leads to a more pronounced decrease in $E_{g}$ compared to doping at the Li site, potentially enhancing conductivity. Similar trends in $E_{g}$ reduction are observed across other L*M*PO_4_ compounds. Conversely, substituting ions with a larger ionic radius than Fe, such as Zn and Cd, causes an increase in $E_{g}$.

## 1. Introduction

Lithium-ion batteries have garnered significant interest for portable devices due to their superior energy density compared to other systems. With a growing demand for rechargeable batteries in electric vehicles, lithium transition metal phosphates, denoted as Li*M*PO_4_ (L*M*PO), where *M* = Fe, Ni, Co, Mn, are being explored as replacements for the prevalent LiCoO_2_ cathodes in lithium-ion cells. L*M*PO batteries find extensive applications in energy storage systems. L*M*PO has emerged as a crucial cathode material for next-generation power lithium-ion battery applications due to its abundant raw materials, eco-friendliness, excellent cycling performance, and safety attributes. However, the commercial utilization of L*M*PO cathode material has been impeded thus far by its low electronic conductivity. Strategies such as ion doping or synthesizing smaller particles have proven effective in enhancing electronic conductivity and reducing the band-gap energy [1,2,3,4,5,6].

The magnetic properties of many compounds of the L*M*PO family have been widely studied, mainly because of their frustration effects. In the present paper, using the s-d model, we show that the magnetic and electronic properties in L*M*PO are connected. This means that the magnetic properties can affect the electronic ones, and vice versa. Zaghib et al. [7] reported that in LiFePO_4_ (LFPO), there is indirect evidence of the existence of small polarons, showing the interplay between the electronic and magnetic properties. Therefore, before delving into methods to enhance conductivity and reduce the band-gap energy, it is crucial to underscore that compounds within the L*M*PO family exhibit antiferromagnetic properties [8,9,10,11,12]. These compounds, comprising corner-sharing MO_6_ octahedra with high-spin $M^{2+}$ ions, exhibit an antiferromagnetic ground state typically below a transition temperature, $T_{N}$, ranging from 30 to 50 K [13]. Notably, weak ferromagnetism has been observed in LiMnPO_4_ (LMPO), as reported by Arcon et al. [14]. The ground state of LFPO is characterized as a collinear antiferromagnet, whereas LMPO exhibits a weak ferromagnetic ground state with a transition temperature, $T_{N}$, of approximately 42 K. LFPO boasts the highest Neel temperature ($T_{N}$ = 50 K), below which it demonstrates antiferromagnetic ordering [15]. These magnetic property disparities stem from differences in electronic structures, which could have implications for the electrochemistry of LFPO and LMPO. Spin-wave dispersions in LFPO’s antiferromagnetic state have been elucidated through a linear spin-wave theory by Li et al. [16]. Additionally, antiferromagnetic behavior was observed in LiCoPO_4_ (LCPO) and LiNiPO_4_ (LNPO) by Vaknin et al. [10], showcasing characteristics akin to weakly coupled two-dimensional Ising antiferromagnets. LNPO exhibits a spontaneous first-order magnetic phase transition. Dai et al. [17] conducted an analysis of LMPO’s spin-exchange interactions utilizing density functional theory (DFT) calculations, shedding further light on their magnetic properties.

Mercier et al. [18] elucidated the intriguing magnetoelectric properties of isostructural transition-metal lithium orthophosphates, denoted as L*M*PO systems. These materials exhibit a significant interplay between their magnetic and electric properties, rendering them promising candidates for advanced functional applications. In parallel investigations, Khrustalyov et al. [9] and Vaknin et al. [10] independently documented the magnetoelectric effect within the antiferromagnetic framework of LNPO. This phenomenon underscores the intricate coupling between magnetic and electric degrees of freedom in these compounds. Moreover, recent investigations by Fogh et al. [19] have provided valuable insights into the magnetic field-induced electric polarization in LFPO, offering a comprehensive examination of this phenomenon across a range of temperatures and applied magnetic field strengths. Notably, their findings unveiled a previously unreported diagonal magnetoelectric tensor element, thus enriching our understanding of the underlying mechanisms governing the magnetoelectric behavior in these systems. Building upon these foundational studies, future research endeavors are poised to delve deeper into the magnetoelectric properties of L*M*PO materials, promising further advancements in the field of multifunctional materials science.

Undoped LFPO typically exhibits semiconductor behavior with a considerable band gap ranging from 3.8 to 4.0 eV, as established by previous research [20]. However, modifications through doping, particularly on the lithium or iron sites, have been demonstrated to reduce the band gap, as observed with dopants such as V [21] or Nb [22]. The introduction of high-valent metal ions, such as Nb^5+^, Al^3+^, Ti^4+^, K^+^, and Na^+^, among others, at the Li site has been shown to enhance the conductivity and hole mobility in LFPO, thereby positively influencing its electrochemical performance [1]. DFT calculations, specifically involving Nb substitution for Fe or Li in LFPO, have elucidated the electronic structure of the material, indicating a decrease in the band gap post-doping, leading to enhanced electronic conductivity [5]. Furthermore, investigations by Gao et al. [23] into Zr and Co co-doped LFPO, utilizing first-principle calculations, revealed a similar trend of a reduced band gap and improved electrochemical properties, underscoring the potential of co-doping strategies in enhancing LFPO performance. Other studies, such as those by Xu et al. [24], have explored the effects of doping on LFPO electronic properties, highlighting the band-gap reduction associated with dopants like Mn at the iron site or Na at the lithium site. Additionally, ion doping with elements such as La, Y, and Na has been reported to decrease the band gap and increase conductivity in LFPO [5,25]. Moreover, research by Zhang et al. [1,26] employed DFT to compute the band gap of LNPO doped with transition metal atoms. Furthermore, investigations conducted by various researchers [27,28,29,30] have explored the impact of substituting Mn in LMPO with Co, Cr, Cu, Fe, Ni, or V on a range of properties, including structural, magnetic, electronic, and conductivity characteristics.

The present study seeks to explore a novel approach by investigating the band-gap energy and conductivity in ion-doped L*M*PO, using, for the first time, a microscopic model and Green’s function theory. This method allows for a more comprehensive understanding of the dynamic behavior of ion-doped L*M*PO, offering insights into their properties across a range of temperatures and excitation conditions. We look for doping ions at the Li or Fe sites that can increase or decrease the magnetization and band-gap energy. Due to the different ionic radii of the doping and host ions, which lead to different strains, the exchange interaction constants in the doped material are modified compared to the undoped one. We explain, on a microscopic level, the doping mechanism. The obtained decrease in the band-gap energy for some doping ions could be used in applications for enhancing the electronic conductivity in L*M*PO_4_ compounds. Moreover, we observe that by doping, for example, with Nb, Ti, or Al ions at the Li site, the magnetization increases, i.e., weak ferromagnetism appears in LiFePO_4_. Unfortunately, there are no experimental data for this statement.

It should be noted that the majority of studies on ion-doped L*M*PO utilize DFT [1,5,20,21,22,23,24,26,27,28]. DFT is a very powerful tool for investigating many-body problems. However, DFT is mostly concerned with ground-state properties at zero temperature. In our approach, we are able to cover the whole temperature regime. It is a finite temperature analysis and includes the entire excitation spectrum. In particular, the method allows us to study the total phase diagram, which is based on the different excitation energies realized in the system. The disadvantage of our approach lies in the consideration of collective properties from the beginning. Our basic quantities are not the naked electrons but the effective spins of the underlying quasi-particles. However, with DFT, all parameters of the system can, at least in principle, be calculated, so we are forced to use additional models to determine these parameters. We are convinced that both approaches, DFT and the Green’s function method, are appropriate and, to a certain extent, can be alternatives for describing many-body systems.

## 2. Model and Method

**Figure 1:**
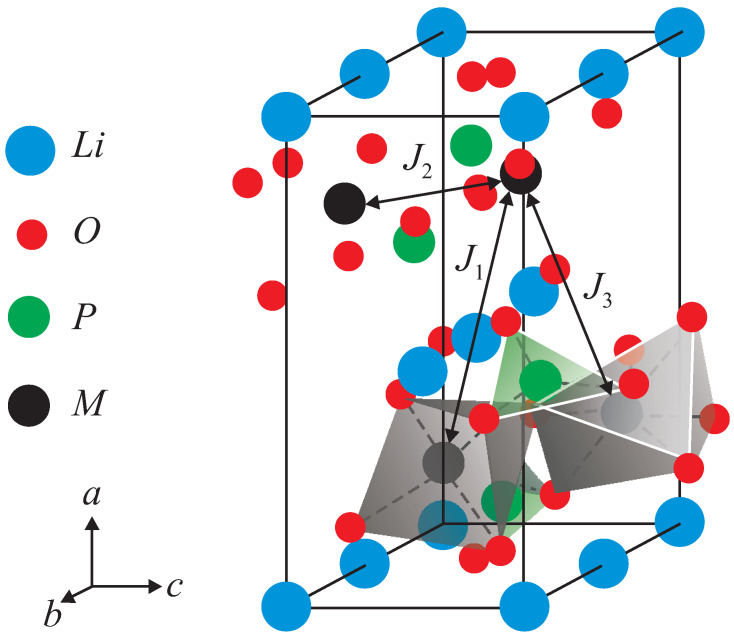
Structure of the unit cell of Li*M*PO_4_.

The olivine-type compounds, Li*M*PO_4_ (*M* = Mn, Fe, Co, Ni), consist of *M*O_4_ layers made up of corner-sharing *M*O_6_ octahedra of high-spin $M^{2+}$ ions (see Figure 1). The magnetic properties, for example, of ion-doped LiFePO_4_ with doping concentration, *x*, are described by the Heisenberg Hamiltonian:

(1) $$H_{sp}=-\frac{1}{2}\sum_{i,j}\left(1-x\right)J_{ij}S_{i}^{Fe}\cdot S_{j}^{Fe}-\frac{1}{2}\sum_{i,j}xJ_{dij}S_{i}^{DI}\cdot S_{j}^{DI}-D_{z}\sum_{i}{\left(S_{i}^{z}\right)}^{2},$$

where $S_{i}$ and its *z* component $S_{i}^{z}$ are the spin operators for the localized Fe^2+^ spins at site *i*. $J_{ij}$ stands for the spin-exchange interactions, including $J_{1}$ and $J_{2}$, which are the in-plane nearest-neighbor (nn) and next-nearest-neighbor (nnn) coupling constants, respectively, as well as $J_{3}$, which is the inter-plane nn coupling constant (see Figure 1). $J_{dij}$ is the exchange interaction constant between the doping ions (DI), *x* is the ion-doping concentration, and $D_{z}$ is the single-site anisotropy parameter of the easy-axis type.

For the investigation of the band-gap energy, we use the s-d model:

(2) $$H_{m}=H_{sp}+H_{el}+H_{sp-el}.$$

$H_{sp}$ is the Heisenberg model of the localized spins, which is described by Equation (1). $H_{el}$ represents the usual Hamiltonian of the conduction band electrons

(3) $$H_{el}=\sum_{ij\sigma }t_{ij}c_{i\sigma }^{+}c_{j\sigma },$$

where $t_{ij}$ is the hopping integral and $c_{i\sigma }^{+}$ and $c_{i\sigma }$ are the Fermi-creation and -annihilation operators.

The Hamiltonian, $H_{sp-el}$, couples the two subsystems—Equations (1) and (3)—by an intra-atomic exchange interaction, $I_{i}$:

(4) $$H_{sp-el}=\sum_{i}I_{i}S_{i}s_{i}.$$

The spin operators, $s_{i}$, of the conduction electrons at site *i* can be expressed as $s_{i}^{+}=c_{i+}^{+}c_{i-}$, $s_{i}^{z}=\left(c_{i+}^{+}c_{i+}-c_{i-}^{+}c_{i-}\right)/2$.

The band-gap energy

(5) $$E_{g}=\epsilon^{+}\left(k=0\right)-\epsilon^{-}\left(k=k_{\sigma }\right)$$

is defined as the energy difference between the valence and conduction bands:

(6) $$\epsilon_{ij}^{\pm }=\epsilon_{ij}-\frac{\sigma }{2}I\langle S^{z}\rangle .$$

The electronic energies are obtained from the Green’s functions $g_{ij\sigma }=\;\ll c_{i\sigma };c_{j\sigma }^{+}\gg$, where σ=±1. $\epsilon_{ij}$ is the conduction band energy in the paramagnetic state, $\langle n_{m\sigma }\rangle$ is the occupation number distribution, and $\langle S^{z}\rangle$ is the magnetization.

The magnetization, *M*, for an arbitrary spin, *S*, is given by

(7) $$M=\langle S^{z}\rangle =\frac{1}{N}\sum_{i}\left[\left(S+0.5\right)\coth\left[\left(S+0.5\right)\beta E_{mi}\right]-0.5\coth\left(0.5\beta E_{mi}\right)\right],$$

where $E_{mi}$ is the spin-wave energy, calculated from the poles of the Green’s function $G_{ij}\left(t\right)=\;\ll S_{i}^{+}\left(t\right);S_{j}^{-}\gg$.

## 3. Numerical Results and Discussion

The numerical calculations are performed in the JAVA programming environment using simple iterative procedures and summation over nearest neighbors with the following model parameters: $J_{1}$ = −0.662 meV, $J_{2}$ = −0.27 meV, $J_{3}$ = 0.021 meV, *D* = −0.37 meV [16], *S* = 2, *t* = 1 eV, and *I* = 0.5 eV.

Let us emphasize that *J* depends on the distance between the spins and on the lattice parameters. Due to the different strains caused by different doping ions, $J_{d}$ in the doped state can be changed compared to the undoped state, *J*; that is, it can differ from *J*.

### 3.1. Dependence of the Magnetization on the Doping with Nb, Ti, and Al at the Li site in LFPO

**Figure 2:**
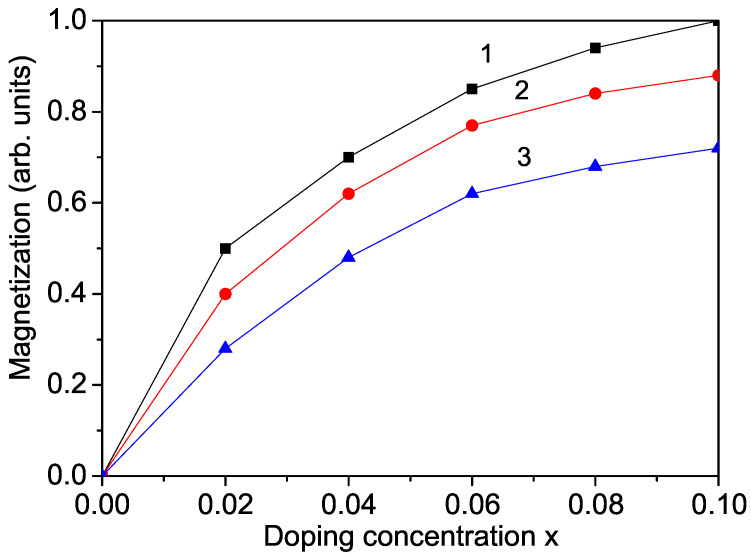
Doping concentration dependence of the magnetization, *M*, in LFPO for different dopants at the Li site: (1) Nb; (2) Ti; (3) Al.

Pure lithium iron phosphate (LFPO) exhibits a substantial band-gap energy of 3.763 eV [21], resulting in low conductivity. To address this limitation and potentially reduce the band-gap energy, we explore the effects of ion doping on LFPO. The calculation of the band-gap energy, $E_{g}$, necessitates knowledge of the magnetization, *M*, as per Equation (6). LFPO, being an antiferromagnetic compound, undergoes initial substitution at the Li site with ions such as Nb^5+^, Ti^4+^, or Al^3+^, possessing smaller ionic radii of 0.64 Å, 0.745 Å, and 0.675 Å, respectively, compared to Li^+^ (0.9 Å). This substitution induces compressive strain, thereby enhancing the exchange interaction constants, $J_{d}$, with $J_{d}$ surpassing *J*. Notably, *J* is contingent upon the spin distance and lattice parameters inversely proportional to it. The magnetization’s dependence on the doping concentration is illustrated in Figure 2, revealing a trend of increasing *M* with higher dopant concentrations. Additionally, the substitution of Li with ions featuring smaller ionic radii than Li results in enhanced *M*, indicating the emergence of weak ferromagnetism upon ion doping. It can be assumed that the electron carrier doping effects in the antiferromagnetic LFPO indicate that a transition from an antiferromagnetic to a weak ferromagnetic phase occurs upon ion doping.

It should be noted that the doping concentration in the experimental works, in general, is small, for example, for Al doping, x = 0.02; for Co, it is 0.03; for Nb, it is 0.01; for Ru, it is 0.05; for Mg, it is 0.1, etc. A high doping concentration would lead to negative effects. With the increase in the dopant content, impurity phases appear. Moreover, excessive ion doping could lead to a decrease in the Fermi energy.

### 3.2. Dependence of the Band-Gap Energy on the Doping with Nb, Ti, and Al at the Li Site in LFPO

**Figure 3:**
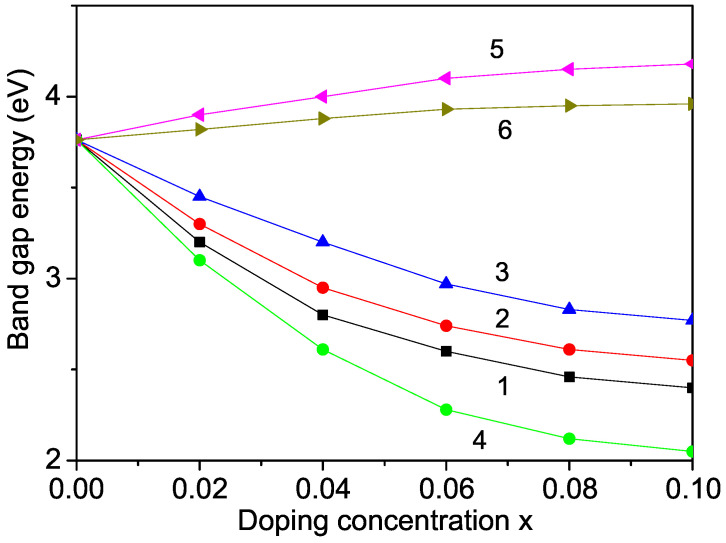
Doping concentration dependence of the band-gap energy in LFPO for different dopants: At the Li site—(1) Nb; (2) Ti; (3) Al—and at the Fe site—(4) Nb; (5) Zn; (6) Cd.

Observing Equation (6), we note a reduction in the band-gap energy, $E_{g}$, upon substituting Nb, Ti, or Al ions at the Li site, as depicted in Figure 3, curves 1–3. These findings align closely with the experimental data from Chung et al. [31] and Zhang et al. [1], suggesting that the decrease in the band-gap energy, $E_{g}$, could potentially enhance LFPO’s conductivity, rendering it a promising cathode material. The introduction of Nb, Ti, or Al ions induces Li vacancies to maintain the charge balance, consequently improving material conductivity. Our model predicts a similar decrease in the band-gap energy, $E_{g}$, upon Mg doping in LFPO at the Li site, consistent with previous research by Yao et al. [32].

Additionally, substitution at the Fe site [22], as exemplified by Ru (*r* = 0.705 Å) or Nb (*r* = 0.64 Å), with smaller ionic radii compared to Fe ions (0.75 Å), induces compressive strain and requires the condition $J_{d}>J$. The decrease in $E_{g}$, as illustrated in Figure 3, curve 4, for Nb-doped LFPO at the Fe site, is more pronounced than that observed for Li ion substitution. This observation is consistent with the findings of previous studies by Gao et al. [33], Zhang et al. [1,22], and Karimzadeh et al. [34], suggesting that Ru or Nb doping at the Fe site effectively enhances LFPO’s electronic conductivity. In summary, doping LFPO with rare-earth ions increases the carrier concentration, shifting the Fermi level and reducing the band-gap energy, $E_{g}$, thereby enhancing conductivity. Co-doping of LFPO with La^3+^ and Y^3+^, as demonstrated by Zhang et al. [5], also improves electronic conductivity, corroborating our model predictions.

### 3.3. Dependence of the Band-Gap Energy on the Doping with Nb, Ti, and Al at the Li Site in LNPO

**Figure 4:**
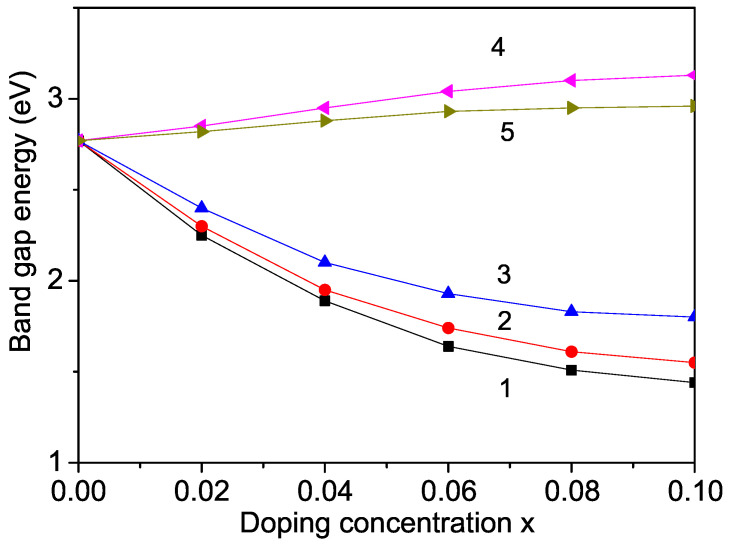
Doping concentration dependence of the band-gap energy in LNPO for different dopants: At the Li site—(1) Nb; (2) Ti; (3) Al—and at the Fe site—(4) Zn; (5) Cd.

Let us broaden our investigation to encompass other members of the Li*M*PO_4_ family and compare their characteristics with those of LFPO. Pure compounds like LMPO and LNPO exhibit relatively high band-gap values: LMPO has a band-gap value of 3.7 eV [35] and LNPO has a band-gap value of 2.77 eV [27]. We once again turn our attention to the band-gap energy, $E_{g}$, a crucial determinant of electronic conductivity, which can be computed from the difference between the conduction band minimums and valence band maximums using Equation (6). Doping with smaller ions such as Nb^5+^, Nd^3+^, Ti^4+^ or Al^3+^ at the Li site in both LNPO and LMPO mirrors the trend observed in LFPO, resulting in a reduction in the band-gap energy, $E_{g}$. This phenomenon is exemplified by the results of Nb, Ti, and Al doping in LNPO, as illustrated in Figure 4, curves 1–3. Correspondingly, Karthickprabhu et al. [36] documented an increase in electronic conductivity upon Nd^3+^ doping in LNPO, indicating a decrease in the band gap, consistent with our model predictions.

Moving forward, we explore the effect of substituting Fe, Ni, or Mn ions with similar doping ions, as in the LFPO case, on the band-gap energy, $E_{g}$. The ionic radii of Ni^2+^ and Mn^2+^ ions are 0.83 Å and 0.97 Å, respectively, whereas that of Fe^2+^ ion is 0.75 Å. Doping with Ru ions at the Ni and Mn sites also results in a reduced band-gap energy, $E_{g}$, in LNPO and LMPO, akin to LFPO. Similarly, substitution with V (*r* = 0.72 Å), Ti (*r* = 0.745 Å), or Cr (*r* = 0.74 Å) ions at the Fe, Ni, or Mn site induces a decrease in $E_{g}$, thereby enhancing the conductivity in all three compounds—LFPO, LNMO, and LMPO. This enhancement in electronic conductivity has been observed in V- and Cr-doped LFPO [21,26], as well as in Cr-doped LMPO [29], corroborating our model predictions.

### 3.4. Dependence of the Band-Gap Energy on the Doping with Zn and Cd on the Ni or Fe Site in LNPO and LFPO

It is crucial to note that not all doping ions induce a decrease in the band-gap energy, $E_{g}$. Taking LNPO as an example, when the Ni ion is substituted with ions like Zn and Cd, with larger ionic radii (0.88 Å and 1.09 Å, respectively) compared to Ni (0.83 Å), it results in a tensile strain, leading to an increase in the lattice parameters. In this scenario, the relationship between the exchange interaction constants in the doped and undoped states follows $J_{d}<J$. As depicted in Figure 4, curves 3 and 4, the band-gap energy, $E_{g}$, increases with higher doping concentrations, a trend corroborated by Zhang et al. [26], who found that transition metal ions (Zn, Cd, and Hg) substituted at the Ni site do not decrease the band-gap energy, $E_{g}$.

Similar behavior is observed in Zn- and Cd-doped LFPO at the Fe site, as shown in Figure 3, curves 3 and 4. This is because the ionic radius of Fe is smaller than that of the substituted ions, resulting in a tensile strain and an increase in the lattice parameters and cell volume. It is noteworthy that the behavior of the band gap strongly depends on the compound’s structure.

Moreover, experimental data on Zn-doped LFPO exhibit discrepancies. Some studies by Shenouda et al. [37], Bilecka et al. [38], and Liu et al. [39] reported an increase in the cell volume, whereas others by Zhao et al. [40] and Yiming et al. [41] suggested a decrease. Unfortunately, experimental data on the band-gap energy or conductivity in ion-doped LMPO are limited.

Observations indicate that the doping effects of Nb, Ti, and Al ions on the Li site in LMPO and LCPO, as well as Zn and Cd ions on the Mn or Co sites, mirror those observed in LFPO and LNPO [29,42]. This consistency underscores the importance of considering the structural effects when assessing doping-induced changes in the band-gap energy across the Li*M*PO_4_ family.

## 4. Conclusions

In summary, our investigation focused on studying the magnetization, *M*, and band-gap energy, $E_{g}$, in ion-doped Li*M*PO_4_ (L*M*PO), where *M* represents various transition metals such as Fe, Ni, Co, and Mn, utilizing a combination of the s-d model and Green’s function theory. We delved into understanding these macroscopic properties from a microscopic perspective. The primary objective of ion doping is to augment the intrinsic electronic conductivity of L*M*PO, although the precise mechanism remains subject to debate within the scientific community. Notably, doping with elements like Nb, Ti, or Al at the Li site in LFPO induces weak ferromagnetism, which intensifies with increasing dopant concentrations. The band gap serves as a pivotal determinant of the electronic conductivity of solid materials, particularly significant in the context of battery materials. Substituting Li with ions possessing smaller ionic radii induces compressive strain, thereby increasing the exchange interaction constants and decreasing the band-gap energy, $E_{g}$. We observed a more pronounced decrease in $E_{g}$ with Nb ion doping at the Fe site compared to the Li site, a trend consistent across various L*M*PO compounds. This reduction in $E_{g}$ signifies a potential enhancement in conductivity, suggesting the potential suitability of L*M*PO as a cathode material. Conversely, substitution with ions of larger ionic radii, such as Zn and Cd, compared to Fe or Ni ions, induces tensile strain, resulting in an increase in $E_{g}$. Overall, our findings align qualitatively with experimental data, further validating our theoretical framework.

Let us emphasize that multi-element co-doping may contribute to better electrochemical performance compared to single doping [1,43]. The substitution on different sites has different effects; for example, on the *M* site, it leads to enhancement of the electronic conductivity, on the Li site it leads to a decrease in the charge transfer resistance, and on the O site, it facilitates the migration of Li ions. Therefore, the combined modification of Li-, *M*-, and O-site co-doping is an effective method to improve the electrochemical properties of the L*M*PO compounds. This problem will be considered in a future paper.

## Data Availability

Derived data supporting the findings of this study are available from the corresponding author upon reasonable request.
